# Automatic Modulation Recognition for Radio Mixed Proximity Sensor Signals Based on a Time-Frequency Image Enhancement Network

**DOI:** 10.3390/s26051677

**Published:** 2026-03-06

**Authors:** Jinyu Zhang, Xiaopeng Yan, Xinhong Hao, Tai An, Erwa Dong, Jian Dai

**Affiliations:** Beijing Institute of Technology, School of Mechatronics Engineering, Beijing 100081, China

**Keywords:** LPI radio frequency proximity sensors, signal recognition, image restoration, deep convolutional neural network

## Abstract

The automatic modulation recognition (AMR) of low probability intercept (LPI) signals has received a considerable amount of interest from many researchers who have done much work on electronic reconnaissance. This recognition technology aims to design a classifier that enables the identification of signals with different modulation types. Based on deep learning models such as a convolutional neural network (CNN), the time-frequency images (TFIs) of the signal can be input to further extract features for classification. To improve recognition accuracy, especially under low signal-to-noise ratios (SNRs), we propose an AMR method for radio frequency proximity sensor signals based on a TFI enhancement network. The TFIs are denoised based on a per-pixel kernel prediction network (KPN), which can improve the quality of TFIs and achieves comparable denoising performance to traditional TFI reconstruction methods (e.g., sparse representation-based methods and low-rank approximation methods), while requiring significantly less computational overhead. The denoised TFIs, with enhanced signal quality and reduced noise, are then fed into the RetinalNet-based classifier as high-quality input features. This enhancement is crucial for the subsequent recognition stage, as it significantly improves the modulation recognition accuracy, particularly under challenging low SNR conditions. Simulation results show that the proposed method can accurately identify the modulation types of different radio frequency proximity sensors that are aliased in the time-frequency domain under low SNRs, and the average recognition accuracy rate of the signal remains above 97% when the signal-to-noise ratio is above −10 dB.

## 1. Introduction

Due to the development of radar technology, low probability intercept (LPI) radar is widely deployed [1,2,3]. The radar has a function to include low effective radiation power and extend the modulation of the radio frequency proximity sensors’ frequency, thus preventing it from being intercepted and identified by controlling the transmitting power; therefore, it has excellent stealth performance [1]. The radar reconnaissance system discovers radio frequency proximity sensors from the complex electromagnetic environment and obtains key parameters of the intercepted radio frequency proximity sensors. In this process, the modulation identification of intercepted LPI radio frequency proximity sensors is a crucial and essential step [2], and the results provide core information for precise interference mitigation [3,4]. At present, automatic modulation recognition (AMR) radio frequency proximity sensors face the following severe challenges: the electromagnetic environment has become more complicated, and features signals with low effective radiation power and various modulation types. Modern information warfare introduces higher requirements for the automation and intelligence of radar radiation source identification [5]. It is more difficult to manually identify the modulation type of an intercepted signal, which cannot adapt to the ever-changing battlefield environment [6]. Therefore, AMR under low signal-to-noise ratios (SNRs) is a problem worthy of deliberation.

Deep learning shows superior capacity compared to traditional machine learning methods [7,8,9], such as Support Vector Machines (SVM) and Random Forests, for learning raw high-dimensional input data. Based on this method, many scholars have studied the automatic identification technology of radio frequency proximity sensors [7,8,9,10,11,12]. Ref. [7] used a deep convolutional neural network (CNN) to recognize modulated signals, and its recognition rate reached 80% under SNRs of −4 dB, while it dropped to about 60% under SNRs of −8 dB. Ref. [8] compared a standard three-layer CNN with the method using higher-order moments and signal statistics to classify 24 radio communication signals. The experimental results show that the application of deep learning can readily classify emitter types with excellent performance. Furthermore, considering the diversity of signal types, there are often multiple numbers and multiple regimes of radio frequency proximity sensors aliasing in the time-frequency domain in the modern electromagnetic environment, which puts forward higher requirements for AMR. Ref. [9] utilized multi-scale convolution and temporal dependency characteristics to recognize waveforms, and the proposed approach demonstrates excellent performance in multi-scale feature extraction from complex temporal patterns. Recent studies have explored time-frequency analysis for signal enhancement [10,11]. Ref. [10] proposed a multi-label framework to identify the modulation types of compound signals. This framework recognizes all compound modulation types through training on limited kinds of signals. Ref. [11] used a multi-instance multi-label learning framework based on a deep convolutional neural network to automatically recognize overlapping LPI radio frequency proximity sensors. It achieves each signal type precisely in the presence of overlapping signals by training a single type of signal.

The above methods only have a preliminary exploration of the AMR of multi-emitter time-frequency aliasing radio frequency proximity sensors, and the recognition accuracy is far lower than the results of single-emitter radio frequency proximity sensors recognition. With the decrease in the signal-to-noise ratio, the time-frequency characteristics of the signal are seriously missing, and the classification accuracy is difficult to meet the needs of use. Recently, researchers have focused their interest on designing a more efficient network to precisely achieve the modulation classification of LPI radio frequency proximity sensors. Only a few studies contemplate improving the quality of data by utilizing image enhancement [12,13,14]. Ref. [12] Introduced a 2-D Wiener filtering to filter the TFI of the signal; the experiments expressed that 2-D Wiener filtering can effectively and essentially improve the classification performance based on DL. Ref. [13] proposed an autonomous feature extraction algorithm to classify low-intercept radar modulations using time-frequency images. In study [13], corrosion and expansion were used to enhance the quality of the time-frequency image and reduce the influence of noise on the signal type recognition task. However, this procedure may account for the loss of signal information in the image details, especially at low signal-to-noise ratios. To obtain pure signal features, ref. [14] ameliorated the TFIs by applying the RGB filter to the images. These articles have verified that the preprocessing of TFIs can improve the classification accuracy. However, the effects of these methods hardly live up to expectations with a low SNR (specifically, when SNR is below −8 dB). When the time-frequency image-based method is used to perform modulation recognition tasks on signals, one of the main factors that restricts the classification accuracy is the quality of the time-frequency image input to the model.

Given the above problems, we propose a TFI denoising method based on a per-pixel kernel prediction network (KPN). In addition, we propose a multi-emitter radio frequency proximity sensor AMR method based on RetinalNet. RetinalNet is used to detect the time-frequency ridges of different signals in the denoised TFI, so as to realize the recognition of each modulation type of different radio frequency proximity sensors with time-frequency aliasing. The results show that when the SNR is in the high regime, which is defined as SNR ≥ −6 dB, the TFI denoising method based on KPN can provide high-quality TFIs for AMR, when the signal is generated under low SNRs, which are defined as −12 dB ≤ SNR < −6 dB, the proposed method can effectively improve the recognition accuracy.

An overview of the proposed method is as follows:

The proposed method consists of three stages. First, the intercepted RF signals are transformed into TFIs using the smoothed pseudo Wigner–Ville distribution (SPWVD). Second, the TFIs are enhanced by a KPN-based denoiser to suppress interference while preserving discriminative structures. Finally, a RetinalNet-based detector performs multi-emitter recognition by localizing and classifying individual signal components in the enhanced TFI.

The remainder of this paper is organized as follows:

Section 2 reviews time-frequency analysis methods commonly used for LPI radar signals. Section 3 presents the TFI denoising approach based on KPN. Section 4 describes the RetinalNet-based AMR method for multi-emitter signal recognition. Section 5 provides simulation results, including ablation studies and performance analysis. Section 6 concludes the paper with a summary and future work directions.

## 2. Time-Frequency Analysis

Time-frequency analysis methods for radar signals can be systematically categorized into two main classes based on their mathematical properties [15,16]. The first class comprises linear methods, including the Short-Time Fourier Transform (STFT) and Wavelet Transform, which superpose time-frequency representations linearly. The second class consists of quadratic methods, which belong to Cohen’s class and include the Wigner–Ville Distribution (WVD) and SPWVD. These methods provide energy density representations in the time-frequency domain and offer superior resolution at the cost of potential cross-term interference.

WVD analyzes the energy density of the signal and has an ideal time-frequency resolution [15,16]. It denotes a quadratic time-frequency representation defined as:

(1) $$WVD_{s}(t,f)=\int_{-\infty }^{\infty }s\left(t+\frac{\tau }{2}\right)s^{*}\left(t-\frac{\tau }{2}\right)e^{-j2\pi f\tau }d\tau$$

where s(t) is the analytic signal, ω is the angular frequency, τ is the lag variable, $s^{*}(\cdot )$ denotes the complex conjugate and $e^{-j2\pi f\tau }$ is the complex exponential kernel that performs the Fourier transform with respect to the lag variable τ.

For single-component chirp-like signals, the WVD provides a highly concentrated quadratic time-frequency representation and satisfies desirable marginal properties. However, since WVD is bilinear, the presence of multiple components inevitably introduces interference (cross-) terms that appear as oscillatory artifacts in the time-frequency plane, which can obscure the true auto-terms and degrade the estimation of instantaneous frequency and energy localization. A more rigorous treatment of these properties and the origin of cross-terms can be found in [16]. To mitigate this issue, the smoothed pseudo Wigner–Ville distribution SPWVD applies separable time-frequency smoothing to suppress cross-terms at the expense of reduced concentration. The SPWVD is given by:

(2) $$\mathrm{SPWVD}(t,f)=\int_{-\infty }^{\infty }\int_{-\infty }^{\infty }s(t-u+\tau /2)s^{*}(t-u-\tau /2)h(\tau )g(u)e^{-j2\pi f\tau }dud\tau$$

where h(τ) and g(u) define the time domain and frequency domain window functions, respectively. And $e^{-j2\pi f\tau }$ is the complex exponential kernel that performs the Fourier transform with respect to the lag variable τ; f denotes the frequency in Hz. After the signal is windowed and smoothed in the time and frequency domains, the cross-term can be well suppressed. Combining the merits and demerits of different time-frequency analysis tools, SPWVD is used to convert signal samples into the entire time-frequency domain in this paper.

## 3. TFI Denoising Method Based on KPN

Traditional threshold-based denoising methods require careful parameter selection and may not generalize well across different SNRs. In contrast, the KPN-based approach learns adaptive filtering strategies directly from data through end-to-end training on TFIs across a wide SNR range, avoiding the threshold selection problem altogether.

**Figure 1:**
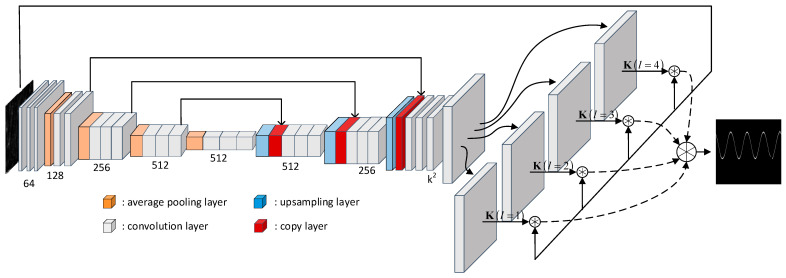
An overview of TFI-DN.

Inspired by recent works on image reconstruction [16,17,18,19], In this section, we present a TFI denoising method based on the Kernel Prediction Network (KPN) to enhance the quality of TFI datasets by effectively removing noise while preserving signal features [20]. The architecture of the TFI denoising network (TFI-DN) is shown in Figure 1. We start by producing a set of multi-dilated pixel-wise image filters with architectures such as U-Net and utilize these filters to handle multi-scale noise streaks of TFIs. The output from each scale channel is merged to obtain the final, denoised image. The TFI-DN obtains higher-level semantic information through multi-layer convolution and generates a pixel-by-pixel filter through up-sampling. Detailed information about the image will be lost in the process of extracting semantic information. The TFI-DN also restores the lost semantics by splicing feature layers to ensure the accuracy of the filter.

In TFI-DN, after the KPN processes input TFIs with noise $I^{\mathrm{TF}}\in {\mathbb{R}}^{H\times W}$, pixel-wise filters are generated to obtain predicted denoising TFIs ${\hat{I}}^{\mathrm{TF}}\in {\mathbb{R}}^{H\times W}$, where H and W are the numbers of discrete bins along the time axis and the frequency axis,

(3) $${\hat{I}}^{\mathrm{TF}}=K*I^{\mathrm{TF}},$$

where $K\in {\mathbb{R}}^{H\times W\times k^{2}}$ is the output tensor produced by the KPN, containing a per-pixel kernel for every spatial location, and ∗ defines the filtering operation.

(4) $$K=\mathrm{KPN}\left(I^{\mathrm{TF}}\right)$$

where $\mathrm{KPN}\left(\cdot \right)$ is a filter prediction network. The KPN has a $k^{2}$ output channel, which is reshaped into a stack of k×k linear filters at each pixel p to closely resemble the architecture in 21. The predicted p-th pixel in ${\hat{I}}^{\mathrm{TF}}$ is

(5) $${\hat{I}}^{\mathrm{TF}}(p)=\sum_{t\in \Omega_{k},q=p+t}K_{p}(t)I^{\mathrm{TF}}(q)$$

where $t=\left(t_{1},t_{2}\right)\in {\mathbb{Z}}^{2},\Omega_{k}=\left\{\left(t_{1},t_{2}\right)\left|\left|t_{1}\right|\leq \frac{k-1}{2},\left|t_{2}\right|\leq \frac{k-1}{2}\right.\right\}$. Specifically, $K_{p}\in R^{k\times k}$ denotes the unique filtering kernel for the pixel at position P, extracted from the tensor K by reshaping the p-th feature vector of size $k^{2}$ into a k×k matrix, which corresponds to the input TFI.

In the deraining task, it is difficult to predict the size of the degraded area in the image. When the degraded area is relatively large, the kernel size will affect the filtering effect. The same situation appears in the TFI denoising process. When the signal-to-noise ratio varies, the intensity and size of the noise and signal on TFIs will be different. Ref. [21] uses a large kernel to obtain information in a larger range and achieve good filtering processing. However, this method leads to a large number of parameters, which increases the time cost. Ref. [22] noted that dilated convolution is used to systematically aggregate multi-scale context information without loss of resolution. In summary, we generate multi-scale filters by expanding the convolution to fuse information of different sizes around the pixels, which adapts to the changes in different scales and positions of the noise. With the offline training of time-frequency images of different signal-to-noise ratios, the KPN can predict spatially varying kernels. This kernel adapts to noise stripes of different thicknesses and intensities while retaining the details and boundary information of the target signal. Equation (5) can be rewritten as

(6) $${\hat{I}}^{\mathrm{TF}}(p)=\sum_{t,q=p+lt}K_{p}(t)I^{\mathrm{TF}}(q)$$

where l is the dilation factor. In this paper, after denoising the input image with 4 filters with $l_{1},l_{2},l_{3},l_{4}=1,2,3,4$, the output results of the 4 channels are fused using the 3×3 convolution to obtain the final denoising time-frequency image.

Following common practice in pixel-wise image restoration, our KPN is implemented as a stack of convolution layers with ReLU, uses average pooling and bilinear upsampling, and is trained with a hybrid objective combining $L_{1}$ and MS-SSIM to balance luminance fidelity and structural consistency [23,24,25].

(7) $$L({\hat{I}}^{\mathrm{TF}},I^{\mathrm{TF}})=\left(1-\lambda \right)\|{\hat{I}}^{\mathrm{TF}}-I^{\mathrm{TF}}{\|}_{1}+\lambda \left(MS-\mathrm{SSIM}({\hat{I}}^{\mathrm{TF}},I^{\mathrm{TF}})\right)$$

Simultaneously, due to the randomness of the position and size of noise in the time-frequency image, we adopt the strategy identical to [25] and fix λ=0.84, which gives the MS-SSIM more weight.

## 4. The AMR Method for Radio Mixed Proximity Sensor Signals Based on a TFI Enhancement RetinalNet

**Figure 2:**
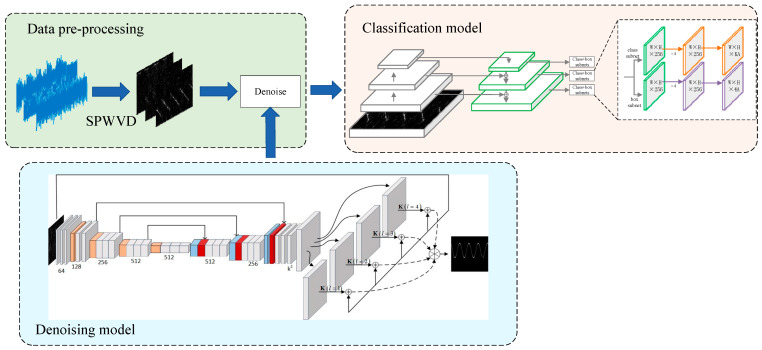
The AMR method for radio mixed proximity sensor signals based on a TFI enhancement RetinalNet.

The process of multi-emitter radio frequency proximity sensors AMR method based on RetinalNet is shown in Figure 2. The intercepted radio signals were processed using a SPWVD to obtain the TFI of the signals for classification. The TFI is denoised by TFI-DN and is used as the input of the classification model.

The RetinalNet is used as the classification model and is trained for multiple rounds offline to obtain the network weight parameters. The classification model is finally tested through the testing set. During offline training, the feature information of the signal is stored in the knowledge base. After the offline training is completed, the intercepted signal can be quickly sorted and identified online. In the process of network training, O’Shea et al. used CNN, VGG [26], ResNet [26], and other network structures for classification, and found that the network structure based on ResNet achieved the best classification effect [27]. Therefore, this paper uses the RetinalNet structure based on ResNet for AMR.

(1) Residual block

Theoretically, the more layers the CNN has and the more complex the model is, the more information it contains and the better the classification effect. However, as the number of network layers continues to deepen, the problem of gradient explosion and disappearance occurs, which makes training and optimization very difficult, and the performance of the classification model begins to decline. The residual block solves the performance degradation problem caused by the increase in depth while increasing the depth of the network.

**Figure 3:**
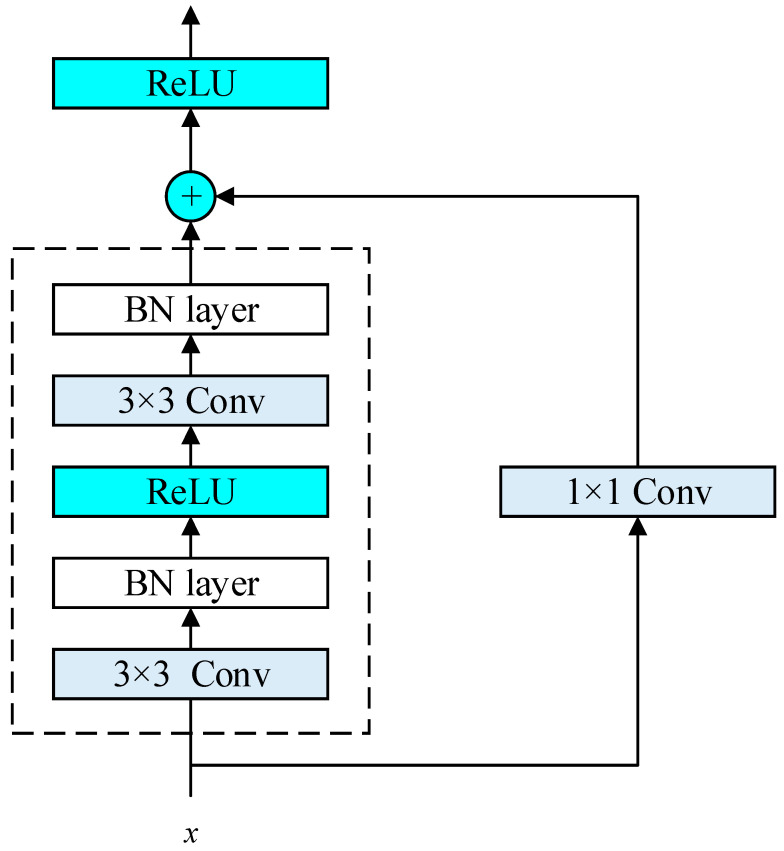
Residual block.

The residual block is shown in Figure 3, which serves as the core feature extraction modules in the RetinalNet backbone architecture. In the overall pipeline (Figure 2), these residual blocks receive the denoised TFI from the KPN module and process it through multiple levels of abstraction. Each residual block contains skip connections that enable effective gradient flow and feature reuse, allowing the network to learn both low-level (e.g., edges, textures) and high-level (e.g., signal patterns, modulation characteristics) features from the TFI. The output of these residual blocks is then fed to the detection head, which identifies and classifies multiple overlapping signals. The block contains two 3 × 3 convolutional layers with the same number of output channels. Each convolutional layer is followed by a batch normalization (BN) layer and a ReLU activation layer. Let x denote the input feature map to the residual block. The block contains two 3×3 convolutional layers. The input x is directly added to the output of the second BN layer via a skip connection, formulated as $F\left(x\right)+x$, before the final ReLU activation layer. A 1 × 1 convolutional layer is introduced to adjust the channel number, so that the input and output dimensions match.

We employ ResNet-50 [28] as the backbone feature extractor in our RetinalNet-based detection framework. ResNet-50 provides an excellent balance between representational capacity and computational efficiency. From the backbone, we extract feature maps at five scales, which are then processed by the feature pyramid network (FPN) to produce multi-scale representations suitable for detecting signals of varying sizes in the time-frequency domain.

(2) FPN

**Figure 4:**
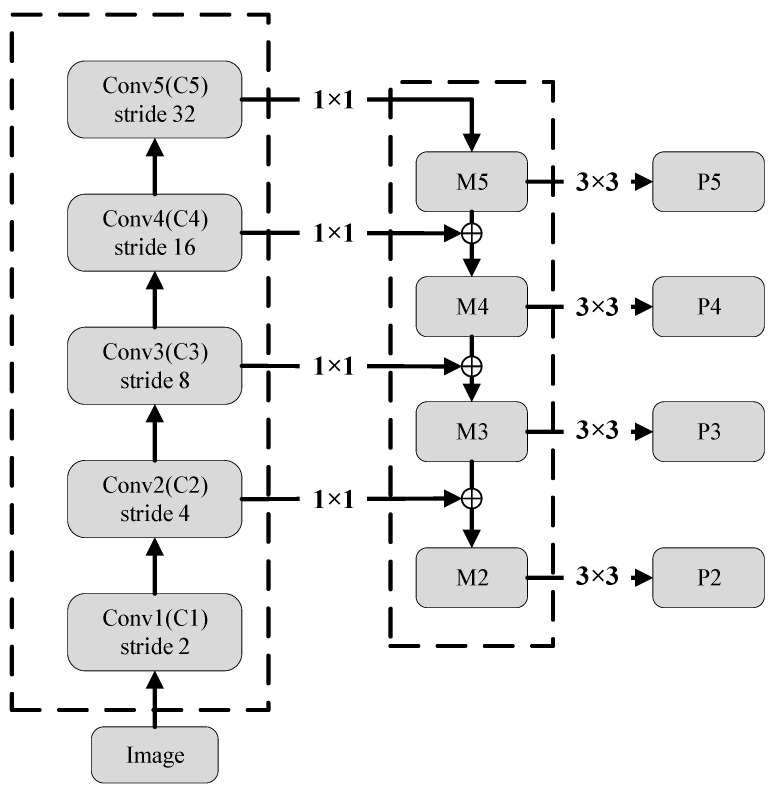
The architecture of the FPN.

FPN enhances the standard convolutional network with top-down paths and lateral connections, effectively constructing a rich multi-scale feature pyramid, where each level of the pyramid can be used to detect objects of different proportions [29]. Features with low levels have small receptive fields and can be used to detect small objects. Features with higher levels have larger receptive fields and can be used to detect large objects. FPN integrates the features of different scales together, making the feature information of each level of the pyramid more abundant, so as to make the extracted features more effective. The FPN used on the RetinalNet network has 5 layers—namely P7, P6, P5, P4, and P3. These five layers are generated from the C3, C4, and C5 three-layer feature maps extracted from the ResNet through horizontal and vertical connections. Only convolutions are included in the FPN model, excluding BN and ReLU layers. Figure 4 shows the architecture of the FPN.

(3) Classification and regression

The detection module consists of two parallel Fully Convolutional Networks (FCNs): a classification subnet and a box regression subnet, that operate on each level of the FPN from P3 to P7. At each spatial location, the network utilizes nine anchors (K=9), configured using three aspect ratios (1:1, 1:2, and 2:1) combined with three scales ($2^{0},2^{1/3}$, and $2^{2/3}$). The classification subnet is tasked with predicting the probability that each anchor contains a signal of a specific modulation type. It takes a 256×H×W feature map from the FPN as input and processes it through four 3×3 convolutional layers, each comprising 256 filters and followed by a ReLU activation. A final 3×3 convolutional layer outputs 63 channels (C×K=7×9), which are subsequently passed through a sigmoid activation function tailored for multi-label classification, ultimately yielding a probability map of shape H×W×63. Parallel to this, the box regression subnet is designed to predict the four bounding box offsets (Δx,Δy,Δw,Δh) for each anchor. It shares an identical architectural structure with the classification subnet up to the final layer. Its final 3×3 convolutional layer produces 36 output channels (4×K=36) and utilizes no activation function since the regression outputs are unbounded, resulting in an offset map of shape H×W×36. Importantly, both the classification and regression subnets employ shared weights across all FPN levels (P3–P7). This specific design choice is based on the observation that the semantic meaning of the features remains consistent across different scales, thereby allowing the network to effectively learn scale-invariant representations.

The bounding box regression branch produces an output of 4 × k, representing the 4 coordinate offsets (x, y, width, height) for each anchor. Translation-invariant anchor boxes are employed, with each scale level having 3 aspect ratios and 3 scales, resulting in k = 9 anchors per location. The number of signals is determined through post-processing of the detection outputs.

RetinalNet is trained with the Focal Loss function [30], thus achieving high detection accuracy. It can weigh each sample separately according to the difficulty of the sample. The weight of the easy sample is low, and the weight of the hard sample is high. The full loss function is as follows.

(8) $$L\left(p_{t}\right)=-\alpha_{t}{\left(1-p_{t}\right)}^{\gamma }\log\left(p_{t}\right)$$

where α is the weighting parameter that belongs to the positive and negative samples, γ is the focusing parameter that controls the down-weighting of easy examples, and $p_{t}\in [0,1]$ is the probability predicted by the model for the target whose true class is 1.

## 5. Simulation Analysis

### 5.1. Experimental Environment and Dataset

All experiments were conducted on a workstation with an NVIDIA GeForce RTX 3080 Ti GPU (12GB GDDR6X) (NVIDIA, Santa Clara, CA, USA) and an Intel Core i9-12900KF CPU (Intel Corporation, Santa Clara, CA, USA), running PyTorch 2.4 with CUDA 12.1 support.

In all experiments, seven modulation-type LPI signals are used: linear frequency modulation (LFM), triangular frequency modulation (Tri), sinusoidal frequency modulation (Sin), sawtooth frequency modulation (Saw), binary phase shift keying (BPSK), Costas codes, and monopulse signals (MP). The definition and equations of all modulations can be found in the publication that describes the principles of radar [30]. To ensure reproducibility, the noise added to the signals is modeled as Additive White Gaussian Noise. These signals are simulated in four repetition periods based on the Monte Carlo method and processed by normalization. Regarding the noise energy control, SNR is controlled as the energy of the signal over the noise and is defined as:

(9) $$\mathrm{SNR}[\mathrm{dB}]=10\;\log_{10}\left(P_{signal}/P_{\mathrm{noise}\;}\right)$$

where $P_{signal}$ is the power of the signal and $P_{\mathrm{noise}\;}$ is the power of the Gaussian noise.

In denoising effect verification, the experimental dataset contains 49,000 TFIs (480×480×1), each dataset has a signal of 1000 TFIs in the SNR range of [−12, −10, −8, −6, −4, −2, 0] dB. In addition, 20% were utilized as the training dataset, and 80% were utilized as the testing dataset.

The networks model training iteration number is 300 and the learning rate is 0.0002. Among them, the multi-core prediction network training model used for filtering is composed of multiple Basic modules. Each Basic module consists of three convolutional networks, with the convolution kernel size being 3, the step size being 1, and the padding being 1. The activation function used in each layer of the network is the ReLU function. After each Basic module, the average pooling method is used to reduce the feature dimension and increase the receptive field of the feature layer. The convolution kernel size of the average pooling is, the step size is 2, and the padding is 0. The parameters of the training model are as shown in Table 1.

In signal identification verification, the experimental dataset contains 91,000 TFIs (480×480×1), each signal has 1000 TFIs in the SNR range of [−12, −11, −10, −9, −8, −7, −6, −5, −4, −3, −2, −1, 0] dB. To compare the classification accuracy of the signal before and after denoising, one ResNet18 was trained by the original TFIs, and the other was trained by the denoising TFIs derived from TFI-DN. The differences between the two ResNet18 datasets lie in the dataset. Eighty percent of the dataset was used as the training dataset, and the remaining 20% was used as the validation dataset.

### 5.2. Denoising Performance

To demonstrate the superiority of our proposed TFI-DN method, we compare it with three classical image denoising baselines: mean filter, median filter, and Wiener filter. The mean, median, and Wiener filters all use a 5 × 5 square window; zero padding is applied at the boundaries, and the Wiener filter estimates noise variance within a 3 × 3 local neighborhood. In addition, in the SPWVD computation, the Gaussian smoothing windows h(τ) and g(u) are used in both the time and frequency directions. The standard deviations are set to 0.5 with normalized time samples and 0.3 with normalized frequency bin, respectively, to balance cross-term suppression and auto-term resolution.

We designed two experiments: denoising effect verification and signal identification verification. In the first part, we employed the three existing methods as our baselines. These three methods are compared with the denoising method proposed in this paper and demonstrate the superiority of our method.

SSIM (structural similarity index) is an index to evaluate the similarity of two images. It measures image similarity from three aspects: image illumination, contrast, and structure [31,32]. The peak signal-to-noise ratio (PSNR) is the most common and widely used evaluation index for images. Its value indicates the degree of distortion. We use SSIM and PSNR as image quality evaluation indicators. A PSNR above 40 dB indicates that the image is very close to the original image; a PSNR of 30~40 dB usually indicates that the distortion of the image is perceptible but acceptable; a PSNR of 20~30 dB indicates poor image quality, and a PSNR below 20 dB is unacceptable.

To ensure a fair comparison, we conducted a preliminary parametric study to optimize the window sizes for the baseline filters. We tested window sizes of 3 × 3, 5 × 5, and 7 × 7. The 5 × 5 window was selected for the mean, median, and Wiener filters as it provided the best trade-off between noise suppression and signal detail preservation. We evaluated window sizes on a validation subset of 1000 TFIs at SNR = −10 dB. The results are shown in Table 2.

**Figure 5:**
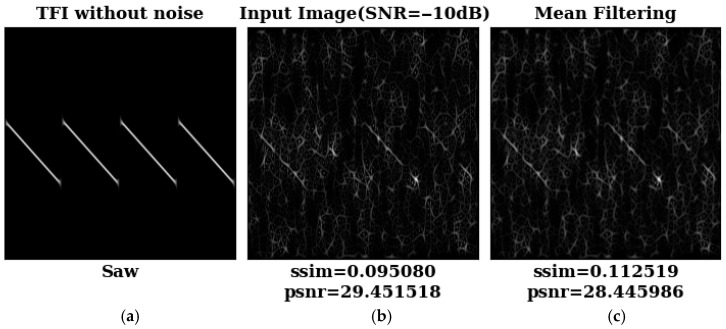

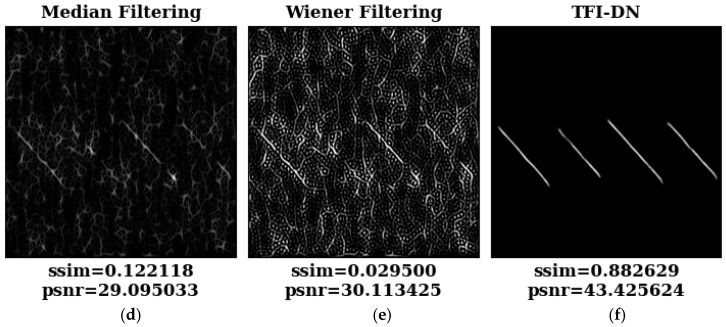
Results before and after denoising for the LFM signal at SNR = −10 dB: (**a**) TFI of LFM without noise; (**b**) TFI at SNR = −10 dB; (**c**) TFI after mean filtering; (**d**) TFI after median filtering; (**e**) TFI after Wiener filtering; (**f**) TFI filtered by our denoising framework.

We first compared the denoising performance of three typical image enhancement methods and TFI-DN. Based on these methods, Figure 5 shows the before and after denoising TFIs of the sawtooth frequency modulation signal at SNR = −10 dB. In Figure 5, Wiener filtering has a better effect than the mean and median filtering denoising TFIs. Meanwhile, the values of SSIM and PSNR reach their maximum when we use our proposed model to denoise the TFIs. This model has the best performance in denoising TFIs, and it greatly improves the quality of the signal dataset.

**Figure 6:**
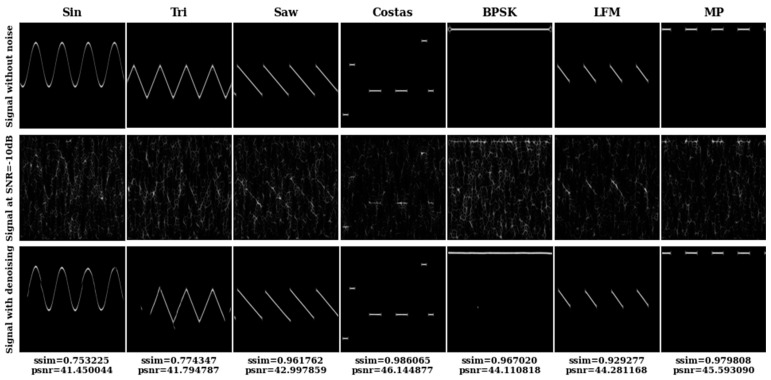
TFIs of 7 radio frequency proximity sensors before and after denoising at SNR = −10 dB.

Based on TFI-DN, we show the denoising results in Figure 4 for the 7 mentioned signals at SNR = −10 dB. In Figure 5, the first row shows the TFIs of the 7 radio frequency proximity sensors without noise. The second row shows the TFIs at SNR = −10 dB, and the third row shows the TFIs produced by our framework, denoising the TFIs of the second row. As shown in Figure 6, the PSNR and SSIM reached particularly high values in our final version. The noise in the picture is well eliminated; in contrast, the signal is well restored. Thus, our contributions are advantageous and effective for improving the quality of datasets. The denoising TFIs obtained by our method have excellent performance, which mainly reflects the ability to reproduce the entire change in the signal in the TFIs, but the details of the signal may produce greater loss and distortion. For example, at SNR = −10 dB, partial information of the triangular frequency modulation signal has been lost, the waveform of the sinusoidal frequency modulation signal has a slight deformation, and the phase information in the binary phase shift keying coded signal has been lost.

**Figure 7:**
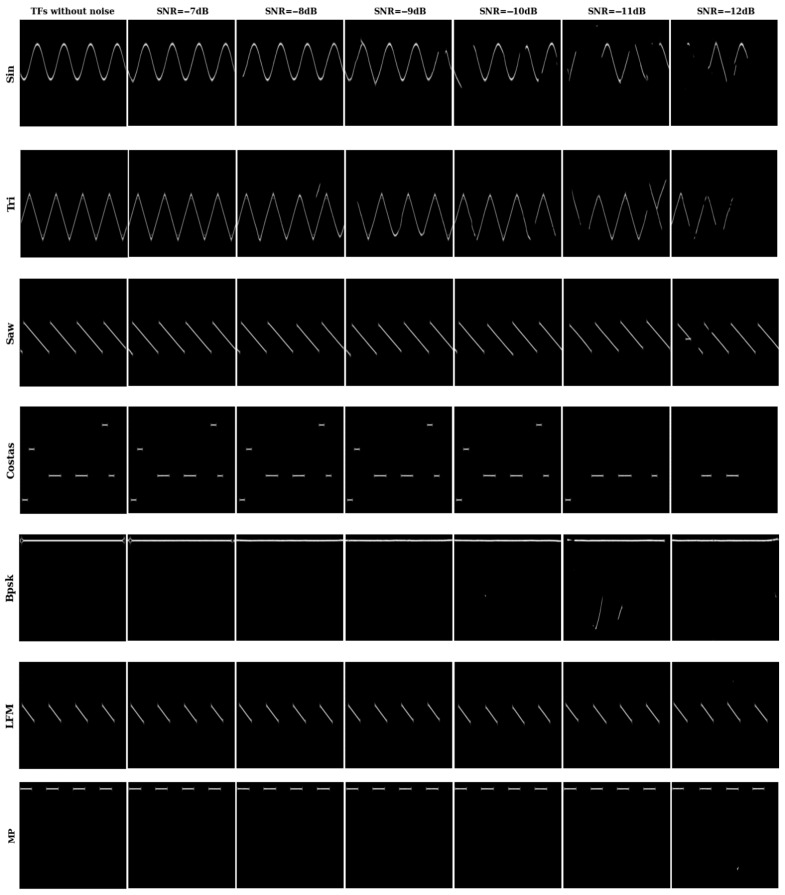
Denoising TFIs of seven different signals at SNR = −7~−12 dB.

To prove that TFI-DN performs well in different signal-to-noise ratios, we show the TFIs of seven radio frequency proximity sensors after denoising. Figure 7 exhibits the denoising TFIs of seven signals in the SNR range of [−7, −8, −9, −10, −11, −12] dB. When the SNR is lower than −10 dB, the denoising performance is obviously degraded, and the restored image has lost a considerable amount of important signal information.

### 5.3. Recognition Performance

To comprehensively evaluate the robustness of our method under varying signal complexity, we define three experimental scenarios based on modulation type diversity. We define the scenarios based on the diversity of modulation types: 1 MT (Single Modulation Type) denotes scenarios where, although there may be multiple emitters, all signals belong to the same modulation class; 2 MT (Dual Modulation Types) denotes scenarios where the concurrent signals are drawn from exactly two different modulation classes, such as LFM mixed with BPSK; and 3 MT (Triple Modulation Types) denotes that concurrent signals are drawn from three different modulation classes, such as the scenarios that LFM, BPSK, and Costas-coded signals appearing simultaneously. In the SNRs of 0 dB~−15 dB, 300 TFIs were generated every 1 dB as the original dataset under the condition of 1 MT to 3 MT, and the number of signals in each TFI ranged from 1 to 6, was not fixed. In total, 80% of the original dataset was used for training the RetinalNet model for recognition and 80% of the original dataset was used for testing the RetinalNet model. To ensure full reproducibility, for each modulation type and each SNR level, we conducted 1000 independent Monte Carlo trials. In each trial, the signal’s parameters—including carrier frequency, modulation frequency, pulse width, frequency deviation, and duty cycle—were independently and randomly sampled from the uniform distributions defined in Table 2. Furthermore, an independent realization of Additive White Gaussian Noise (AWGN) was generated and superimposed on the signal to exactly match the designated SNR. Each simulated signal spans four pulse repetition intervals (PRIs) and is subsequently normalized in amplitude before being transformed into a TFI. The parameter range of the signals are shown in Table 3. All signals are recognized after down-conversion processing and the intermediate frequency variation range is 700~900 MHz.

The number of iterations of the RetinalNet model is 100, and the learning rate is 0.0001. In the 40th and 53rd rounds, the learning rate becomes one-tenth and one-hundredth of the original.

**Figure 8:**
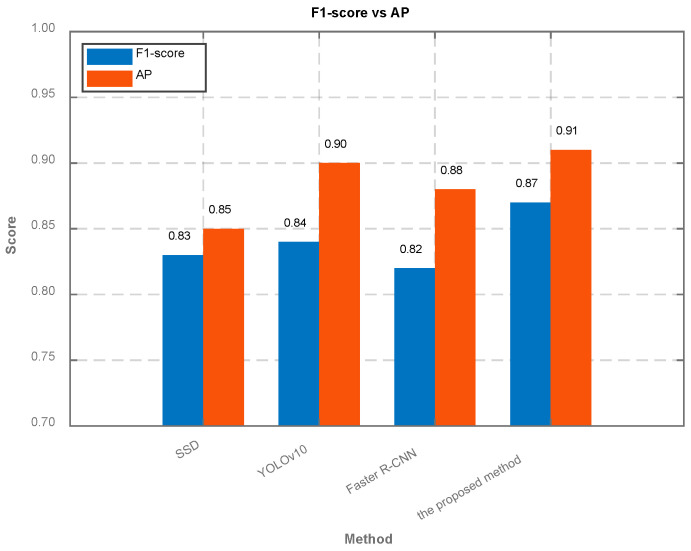
The average recognition accuracy of different algorithms for object detection.

To ensure a rigorous and fair comparison between different object detection architectures, we followed a strictly controlled experimental protocol. Under the SNR of −10 dB, we compared our proposed method with three baseline object detection algorithms: SSD [33], YOLOv10 [34], and Faster R-CNN [35], as shown in Figure 8. The controlled variables are as follows: (1) Input data: All baseline models received the exact same preprocessed TFIs denoised by our KPN module. (2) Data split: 80% of the dataset are randomly sampled as the training set, and 20% of the dataset are randomly sampled as the validation set. (3) Training protocol: all models are trained from scratch without pre-trained weights, and the number of epochs are the same. (4) Evaluation metrics: the AP are at IoU threshold 0.5, and the F1-score are the same at confidence threshold 0.5.

**Figure 9:**
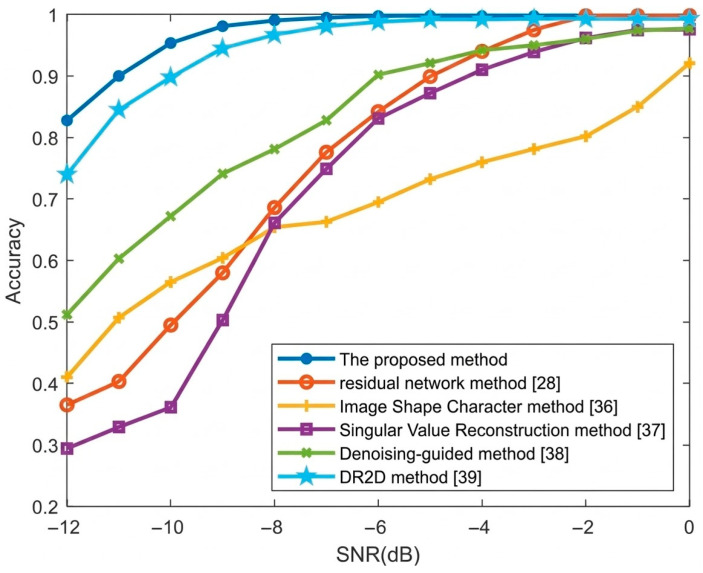
The average recognition accuracy of the seven types of radio frequency proximity sensors.

Figure 9 illustrates the average recognition accuracy of the seven types of radio frequency proximity sensors under low SNRs after using different classification models. The recognition rate curves are shown in Figure 9 and Table 4. Compared with references [28,36,37,38,39] the denoising model proposed in this paper can greatly improve the recognition rate of signal modulation types. Under the SNR of −10 dB, the recognition accuracy of seven radio frequency proximity sensors can reach 95.3%.

**Figure 10:**
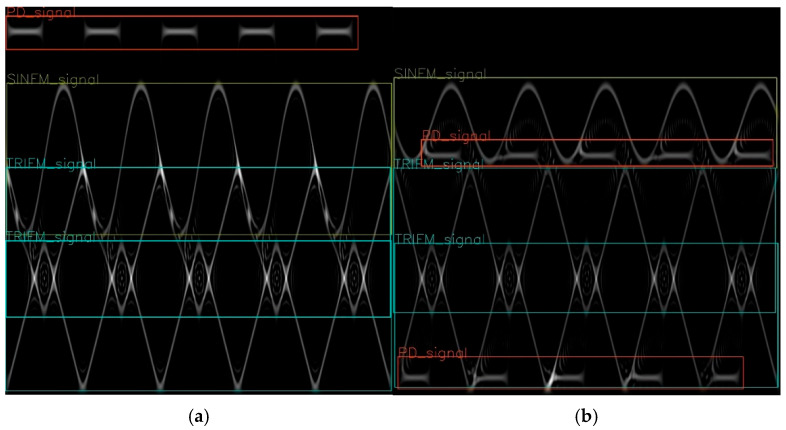
The recognition result of radio frequency proximity sensors. (**a**) Overlapping frequency modulation signals and (**b**) overlapping frequency modulation and pulse signals.

Figure 10a shows the recognition result of one SINFM signal and two TRIFM signals. Figure 10b shows the recognition result with two PD signals, one SINFM signal, and two TRIFM signals. It can be seen that through this model, not only can the modulation type be accurately classified, but the number of signals can also be marked.

**Figure 11:**
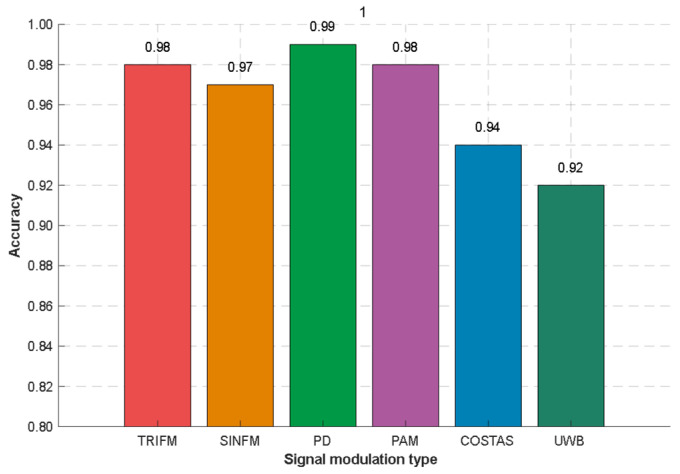
The recognition accuracy for the number of signals in the condition of 1 MT.

When the combination of modulation types is 1 MT and the number of radio frequency proximity sensors is not fixed, the number of radio frequency proximity sensors is recognized under the SNR of −10 dB. According to the results in Figure 11, the recognition accuracy for the number of signals can reach more than 92% for different modulation types.

**Figure 12:**
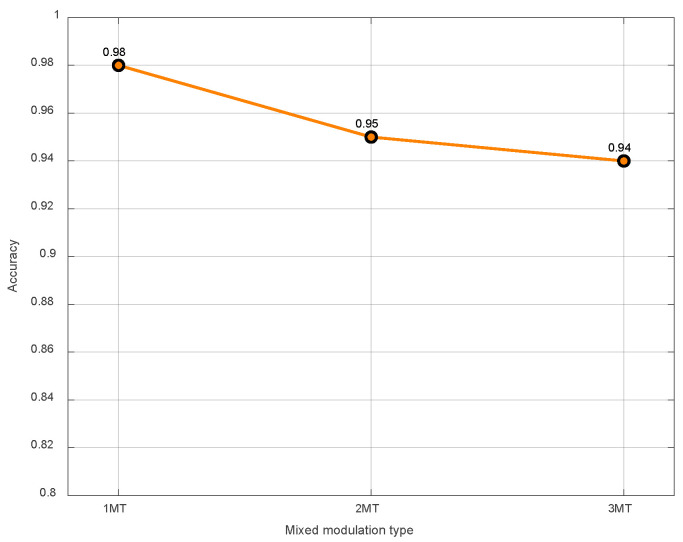
The recognition accuracy for the number of signals in the conditions of 1~3 MT.

When the combination of modulation types is 1~3 MT, and the number of radio frequency proximity sensors is not fixed, the number of radio frequency proximity sensors is recognized under the SNR of −10 dB. According to the results in Figure 12, when the TFI contains multiple modulation types of signals, the average recognition accuracy for the number of signals can reach more than 94%.

**Figure 13:**
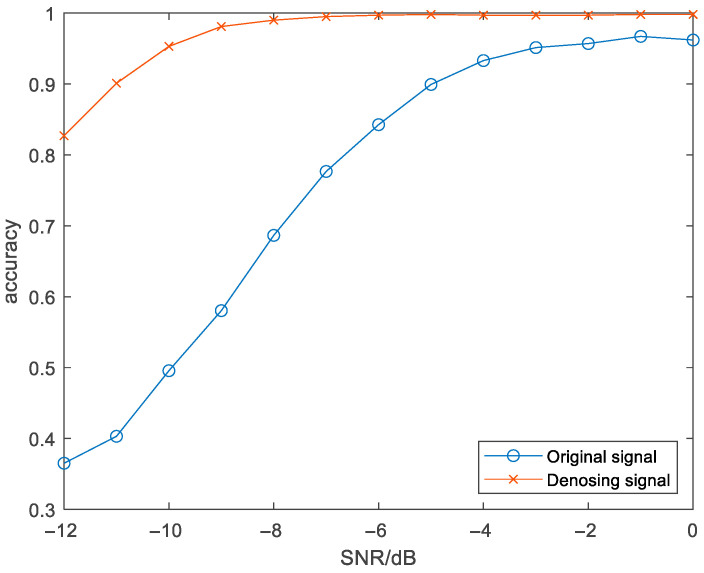
The recognition accuracy for the number of signals under SNRs of 0~−12 dB.

As can be seen from Figure 13, when the signal-to-noise ratio is above −10 dB, the recognition accuracy rate of the undenoised signal is less than 50%. After noise reduction through the noise reduction model we proposed, the average recognition accuracy rate of the signal remained above 97%.

Moreover, the model achieves an average inference speed of 5.3 FPS on the RTX 3080 Ti platform, which corresponds to 189 milliseconds required to complete each AMR process. This inference capability enables practical deployment in reconnaissance applications, allowing intercepted signals to be processed with sufficient speed to support timely threat assessment and tactical decision-making.

### 5.4. Ablation Study

To evaluate the contribution of each component in our proposed method, we designed five ablation configurations with progressive feature additions. Configuration A serves as the baseline using a standard 3-layer CNN classifier directly on raw signals without time-frequency transformation, denoising, or multi-emitter detection capabilities. Configuration B incorporates the SPWVD to transform signals into time-frequency images while retaining the standard CNN classifier. Configuration C adds the KPN denoising module to process SPWVD TFIs before classification. Configuration D replaces KPN with an alternative CNN-based denoiser for comparative analysis of denoising effectiveness. Configuration E represents our complete proposed method, integrating SPWVD transformation, KPN denoising, and the RetinalNet classifier for multi-emitter detection, thus demonstrating the full pipeline’s capabilities. This progressive configuration design allows us to isolate and quantify the individual contributions of time-frequency analysis, denoising, and the object detection framework to the overall recognition performance.

The experimental results, summarized in Table 5, demonstrate the incremental performance improvements achieved by each component. Comparing Configuration A with 0.623 accuracy at negative 10 dB SNR and Configuration B with 0.687 accuracy reveals that time-frequency transformation via SPWVD contributes a 6.4% accuracy improvement by extracting discriminative time-frequency features from raw signals. The integration of KPN denoising in Configuration C yields a substantial 14.5% accuracy gain over Configuration B, elevating performance from 0.687 to 0.832 at negative 10 dB SNR, which underscores the critical importance of high-quality TFIs for reliable recognition under low SNR conditions. Notably, Configuration C outperforms Configuration D with 0.832 versus 0.785 accuracy at negative 10 dB, confirming that our KPN-based denoising approach is more effective than alternative CNN denoisers in preserving signal characteristics while suppressing noise. The complete pipeline in Configuration E achieves the highest accuracy of 0.953 at negative 10 dB SNR, representing a 12.1% improvement over Configuration C, which validates the superiority of the RetinalNet framework for multi-emitter detection tasks compared to traditional single-label CNN classifiers. These results collectively demonstrate that each component, specifically time-frequency analysis, KPN denoising, and object detection architecture, makes essential and complementary contributions to the overall system performance.

## 6. Conclusions

This paper treats the time-frequency image denoising part of the signal classification task as an image restoration task. Unlike current popularly designed high-performance classifiers, we propose a method to increase the classification accuracy by improving the quality of the dataset. When the classifier is fixed, an ideal classification effect can be achieved. In the experiment, we compared the performance between classic noise reduction methods and TFI-DN. According to the denoising results, the merits of our method in improving the dataset are expressed. We used ResNet as the classifier to recognize the signal set before and after denoising. Our method can improve the quality of the dataset and obtain good results in signal classification tasks. The proposed method of first denoising and subsequently classifying according to the requirements is of great help to practical applications. This method eliminates the noise in TFIs and repairs the signal. The classifier can be designed according to actual requirements. At different signal-to-noise ratios, especially when the amount of available data of the intercepted LPI radio frequency proximity sensors is relatively small, the quality of the dataset is particularly important.

Future research of the article will focus on four aspects. First, the final design and debugging of the model should be adjusted according to the signals captured in the real environment. Second, combining denoising with classification tasks is important and necessary, especially in practice. The one-stage framework is more efficient and takes less time. Third, when the intercepted signals contain no trained modulation type, extracting new signals from the noise can be carefully considered. Finally, the current simulation employs a simplified signal generation model for multi-emitter environments. Future work will incorporate more sophisticated radio frequency interference modeling including nonlinear effects such as intermodulation, cross-modulation, and co-channel interference to enhance the practical applicability of the proposed method.

## Figures and Tables

**Table 1:** Model parameters.

Modules	Input Dimension	Output Dimension
Input	480 × 480 × 1	480 × 480 × 1
Basic1	480 × 480 × 1	480 × 480 × 64
AveragePooling2D	480 × 480 × 64	240 × 240 × 64
Basic2	240 × 240 × 64	240 × 240 × 128
AveragePooling2D	240 × 240 × 128	120 × 120 × 128
Basic3	120 × 120 × 128	120 × 120 × 256
AveragePooling2D	120 × 120 × 256	60 × 60 × 256
Basic4	60 × 60 × 256	60 × 60 × 512
AveragePooling2D	60 × 60 × 512	30 × 30 × 512
Basic5	30 × 30 × 512	30 × 30 × 512
Interpolate	30 × 30 × 512	60 × 60 × 512
Basic6	60 × 60 × (512 + 512)	60 × 60 × 512
Interpolate	60 × 60 × 512	120 × 120 × 512
Basic7	120 × 120 × (256 + 512)	120 × 120 × 256
Interpolate	120 × 120 × 256	240 × 240 × 128
Basic8	240 × 240 × (256 + 128)	240 × 240 × (3 × 3)
Interpolate	240 × 240 × (3 × 3)	480 × 480 × (3 × 3)
Filter core	480 × 480 × (3 × 3)	480 × 480 × (3 × 3)

**Table 2:** Window sizes quality comparison.

Window Size	Mean Filter (PSNR)	Median Filter (PSNR)	Wiener Filter (PSNR)	Visual Quality
3 × 3	29.34 dB	29.21 dB	29.92 dB	Under-smoothed
5 × 5	29.65 dB	29.58 dB	30.41 dB	Best balance
7 × 7	29.02 dB	28.87 dB	29.55 dB	Over-smoothed

**Table 3:** The parameter range of six modulation types of radio frequency proximity sensors.

NO.	Modulation Type	CF/GHz	MF/KHz	PW/ns	FD/MHz	DC
1	TRIFM	[9.94, 9.99]	[200, 400]	-	[40, 80]	-
2	PD	[9.99, 10.01]	-	[50, 100]	-	[1, 5]%
3	PPAM	[10.01, 10.05]	-	[50, 100]	-	[1, 5]%
4	SINFM	[3.55, 3.6]	[200, 400]	-	[40, 80]	-
5	UWB	-	-	[1, 2]	-	[0.1, 0.2] %
6	COSTAS	[18.00, 21.45]	[200, 400]	-	[40, 80]	-

**Table 4:** The recognition accuracy of different AMR models with partial SNRs.

SNR (dB)	−12	−11	−10	−9	−8	−7	−6	−5	−4	−3	−2	−1	0
Proposed	0.827	0.901	0.953	0.981	0.990	0.995	0.997	0.998	0.997	0.996	0.996	0.998	0.998
Resnet [28]	0.365	0.403	0.495	0.580	0.686	0.776	0.842	0.899	0.940	0.975	0.998	0.998	0.998
Image shape [36]	0.410	0.507	0.565	0.604	0.654	0.663	0.695	0.732	0.760	0.781	0.802	0.850	0.921
SVR [37]	0.294	0.329	0.361	0.503	0.661	0.749	0.831	0.872	0.910	0.939	0.962	0.975	0.976
Denoising-guided [38]	0.512	0.603	0.672	0.741	0.781	0.828	0.902	0.921	0.942	0.950	0.960	0.974	0.977
DR2D [39]	0.740	0.845	0.898	0.945	0.967	0.981	0.988	0.992	0.992	0.993	0.993	0.993	0.993

**Table 5:** Ablation study results with partial SNRs.

Configuration	SPWVD	Denoising	Classifier	Acc −12 dB	Acc −10 dB	Acc −6 dB	Acc −0 dB
A: Baseline	×	×	CNN	0.452	0.623	0.789	0.887
B: +SPWVD	✓	×	CNN	0.528	0.687	0.816	0.913
C: +KPN	✓	KPN	CNN	0.723	0.832	0.945	0.968
D: +CNN Den	✓	CNN	CNN	0.684	0.785	0.901	0.934
E: Full	✓	KPN	RetinalNet	0.827	0.953	0.997	0.998

The cross symbol (×) indicates the module is not included, while the check mark (✓) indicates it is included.

## Data Availability

The original contributions presented in this study are included in the article. Further inquiries can be directed to the corresponding authors.
